# Using ultrasound to assess the thickness of the transversus abdominis in a sling exercise

**DOI:** 10.1186/s12891-015-0674-3

**Published:** 2015-08-19

**Authors:** Jörn Lükens, Kim J. Boström, Christian Puta, Tobias L. Schulte, Heiko Wagner

**Affiliations:** Department of Motion Science, Westfälische Wilhelms-Universität Münster, Horstmarer Landweg 62b, D-48149 Münster, Germany; Department of Sports Medicine and Health Promotion, Friedrich-Schiller-University Jena, Wöllnitzer Strasse 42, D-07740 Jena, Germany; Department of Orthopedics and Tumor Orthopedics, University Hospital Münster, Albert-Schweitzer-Campus 1, D-48149 Münster, Germany

**Keywords:** Transversus abdominis, Sling system, Ultrasound method, Measurement method

## Abstract

**Background:**

Activation of the deep stabilizing trunk muscle transversus abdominis (TrA) is important for trunk stabilization and spine stability. Sling exercises are used for the activation of trunk muscles, therefore we determined the thickness of the TrA in a standardized sling exercise in comparison to rest and abdominal press. Furthermore we propose a standardized measurement method, which can be used to compare relative muscle thickness levels in different exercises.

**Methods:**

The main objective of the study was to assess and to compare the thickness of the TrA during different conditions; resting condition, sling exercise condition (non-voluntary contraction), and abdominal press condition (voluntary contraction) using a non-invasive ultrasound-based measurement method.

Ultrasound measurement (USM; 8.9 MHz, B-mode) was employed to measure the thickness of the TrA in twenty healthy volunteers (13 m, 7 f), each one measured three times with breaks of 48 h. On each measurement day, the subjects were measured on three different conditions: resting condition (RC), sling condition (SC), and abdominal press condition (APC). The USM images were analyzed using a custom-made MatLab script, to determine the thickness of the TrA.

**Results:**

A two-way repeated-measurements ANOVA was performed with a significant effect of the factor condition [F(2,38) = 47.82, *p* < 0.0001, η^2^ = 0.72], no significant effect of the factor time [F(2.38) = 2.45, p = 0.1, η^2^ = 0.11], and no significant interaction effect [F(4,76) = 0.315, p = 0.867, η^2^ = 0.02]. Statistically corrected post-hoc t-tests revealed significant differences in TrA thickness showing that RC < SC (*p* < 0.001; η^2^ = 0.19; d = 0.96), SC < APC (*p* < 0.0001; η^2^ = 0.23; d = 1.10), RC < APC (*p* < 0.0001; η^2^ = 0.53; d = 2.11). As for the test-retest reliability the intra-class correlation coefficient (ICC) yielded a value of 0.71, 0.54, and 0.29, on the conditions RC, SC, and APC, respectively.

**Conclusions:**

We showed that the investigated sling exercise can be used to significantly increase the TrA thickness, and that the TrA thickness was significantly different on the three conditions (RC, SC, APC) using the ultrasound-based method.

## Background

The transversus abdominis (TrA) is one of the most important muscles for the stability of the lumbo-pelvic area, and is therefore considered to be of central importance for the treatment of low back pain [1–3]. There are few methods to assess the activity of the TrA, such as intramuscular electromyography (iEMG), which is invasive, and muscle cross-section measurements from magnetic resonance imaging (MRI), which is expensive. Another method is to derive muscle activity from muscle thickness [4–7] using ultrasound measurement (USM), which is a relatively cheap, non-invasive, and an easy-to-use method entailing no health risk for the subject [7].

In the last decades, sling exercise therapy became more popular. Ljunggren *et al.* [8] performed a long-term intervention. At first, the patients underwent general physiotherapy, subsequently they carried out a sling exercise training at home with a compliance of 83 %. This intervention led to a decrease of absenteeism by 75-83 % as compared to the year before. Stuge *et al.* [9] also find a positive effect for the treatment of low back pain after pregnancy using sling exercises. Vasseljen *et al.* [10] focused on the correlation of the increase in TrA thickness and the level of low back pain. The pain reduction was correlated with an increased activation of the TrA and a decreased activation of the *obliquus internus abdominis*. Lee *et al.* [11] published a systematic review on the effectiveness of sling exercise therapies for the treatment of low back pain, and reported significant differences in the activation of the abdominal muscles, but no significant differences in pain reduction and disability against other forms of treatment. The sling condition used in our study was intended to represent a standardized exercise to activate the TrA, bearing in mind the above evidences concerning the treatment of low back pain.

The objective of the present study was to assess and compare the activation of the TrA during rest, during exercise in a sling system, and during abdominal press, using a non-invasive ultrasound-based measurement method.

## Methods

### Participants

The study started with 21 healthy volunteers with a body mass index (BMI) between 18 and 28 kg × m^−2^ and without acute back pain for at least 3 months. One subject was excluded because he did not mention a back pain disorder three weeks before the examination. There were 13 male subjects with a mean BMI of 23.7 ± 1.3 kg m^−2^ and a mean age of 25.9 ± 3.1 years, and there were seven female subjects with a mean BMI of 21.1 ± 2.2 kg m^2^ and a mean age of 27.3 ± 2.3 years (Table 1).

**Table 1:** Subjects

Subject	Age [yrs]	Height [m]	Weight [kg]	BMI [kg m^−2^]
1	29	1,7	60	20,76
2	26	1,8	80	24,69
3	25	1,69	59	20,66
4	25	1,79	77	24,03
5	27	1,89	80	22,4
6	26	1,68	56	19,84
7	25	1,78	69	21,78
8	29	1,65	49	18
9	23	1,78	75	23,67
10	32	1,84	68	20,09
11	28	1,74	75	24,77
12	27	1,72	71	24
13	19	1,83	83	24,78
14	26	1,83	79	23,59
15	29	1,85	83	24,25
16	26	1,69	68	23,81
17	24	1,8	78	24,07
18	25	1,84	79	23,33
19	25	1,76	74	23,89
20	30	1,61	64	24,69

Indicated are their age in years, height in m, weight in kg, BMI in kg m^−2^

The study was approved by the University of Münster’s Ethics Committee. Each subject gave written consent to the terms of participation and they were informed to can stop participation at any time.

### Ultrasound Measurement (USM)

**Fig. 1:**
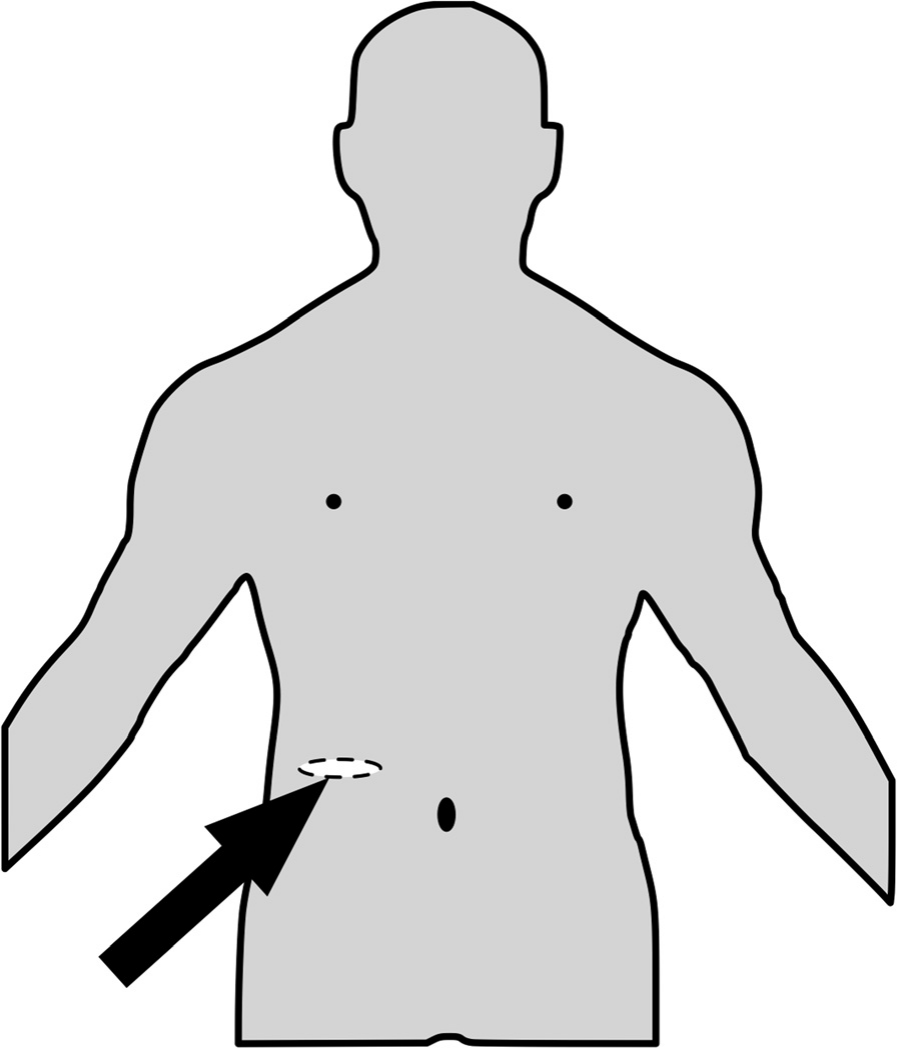
Transducer location. Location of the ultrasound measurement (dashed region). The transducer was positioned horizontally between the lowest rib and the anterior iliac crest

A Siemens Acuson X300 Premium Edition (Erlangen, Germany) was used for the ultrasonic images (B-mode, 8.9 MHz). The linear transducer (VF13-5) was positioned on the right side of the abdomen between the iliac crest and the lowest rib (cf. [4], Fig. 1).

### Experimental setting

All ultrasound measurements have been carried out by one author of this study (JL). During measurement, the subjects were instructed to breathe in deeply, then breathe out about half of the lung volume, and hold their breath on until the image is taken. This instruction was given to prevent stretching or contraction of abdominal muscles. On maximum inhalation, the *diaphragma thoracolumbale* contracts and compresses the *viscerum* [1], the abdomen is enlarged, and the abdominal muscles are stretched and therefore get thinner [12]. On maximum exhalation, the abdominal muscles are activated to press out the air [12]. To determine the sectional area of the TrA, the digital image was analyzed with a custom-made MatLab script (see section Data analysis). The USM took place in three different measurement conditions, and was repeated three times with an intermediate interval of 48 h. The first image was taken in supine with no voluntary activation of the abdominal muscles (resting condition, RC). The second image was taken in supine with maximum voluntary contraction of abdominal muscles, and without movement of the body (abdominal press condition, APC). The third image was taken in prone, suspended in a sling system (sling condition, SC).

### Sling system

**Fig. 2:**
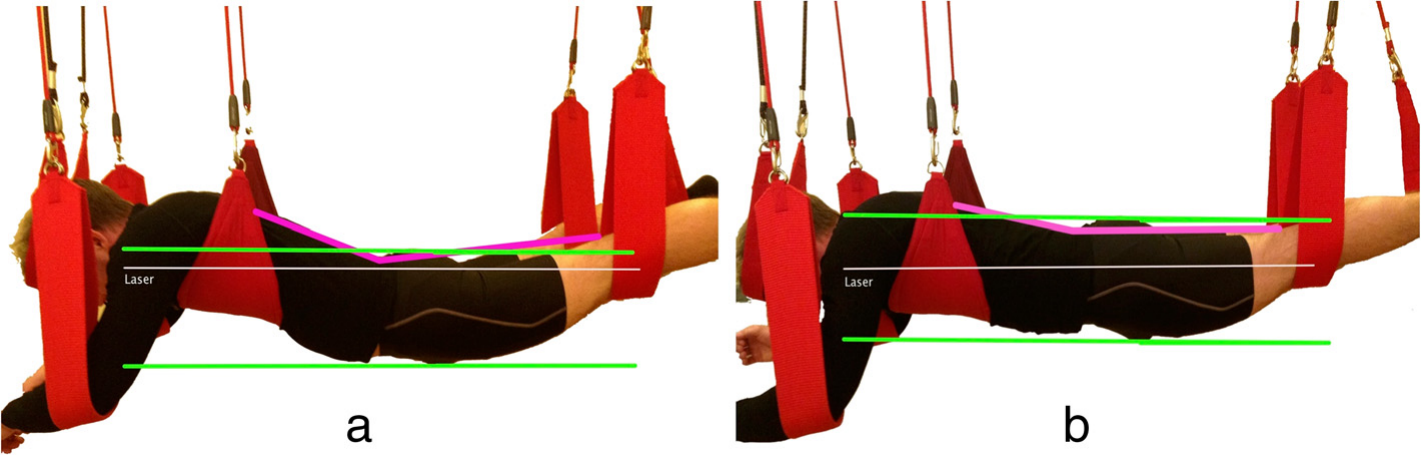
Sling system. Positioning of subjects in the sling system for measurement on the SC. A laser beam (white line) was used to adjust and control the position of the subject. The upper and lower green lines help to identify the ventral and dorsal side of the pelvis, respectively. The purple line helps to estimate the angle between the lower extremities and the trunk. **a** Resting position in the slings: The centers of the lig. fibulare collaterale and of the caput humeri are in line with the beam. **b** SC: The centers of the lig. fibulare collaterale, of the trochanter major, and of the caput humeri are in line with the beam (pelvic lift)

A RedCord® (Staubo, Norway) sling system was used consisting of a stand with three traverses and eight slings (Fig. 2). The arms of the subject were placed into two slings, the head into one sling, the chest into a wider sling, and the knees and ankle joints were placed into two slings per leg.

A height-adjustable bench was lowered until the subject hung with their whole body weight in the sling system (Fig. 2a). Then the subject was asked to close their legs without applying too much pressure. The investigator helped them to bring their pelvis in a neutral position (neutral nutation [13]) between knee and shoulder, with his hands on the subject’s pelvis until they held the target position (Fig. 2b). A Bosch Quigo® (Munich, Germany) laser was used to project a beam on the lateral left side of the subject to control their position. The neutral position was achieved when the trochanter major was in line with the beam, and the lordosis of the lower spine was slightly reduced (Fig. 2b). Then they were instructed to hold the position without concentrating on contracting specific muscles. The subject had to hold the position until fatigue occurred or they could not stay in the target position, in which case the subject got a two minutes break.

### Data analysis

To determine TrA thickness, the sectional area of its two-dimensional representation on the ultrasound image was measured in pixel units (pxu, distance between the centers of two neighboring pixels). A custom-made MatLab script was used to determine the sectional area of a polygon defined by points manually set along the superficial and deep fascia of the TrA and their intersection point (IP), which marked the transition of the muscle into its aponeurosis (Fig. 3.1). The software drew a straight line crossing the IP and running parallel to the main axis of the polygon (Fig. 3.1, line a). At a distance of 250 pxu from the IP to the lateral border of the TrA, a straight line perpendicular to the former line was drawn (Fig. 3.2, line b). The polygon was cut off at this line because the ultrasound image did not show more of the muscle border of the TrA, and the area in the remaining polygon was calculated (Fig. 3.2, grey band area), yielding the final measure for the sectional area of the TrA. The same procedure was repeated for each subject, on each condition, and on each measurement day, with the values of the three measurement days being averaged (cf. [14]).

**Fig. 3:**
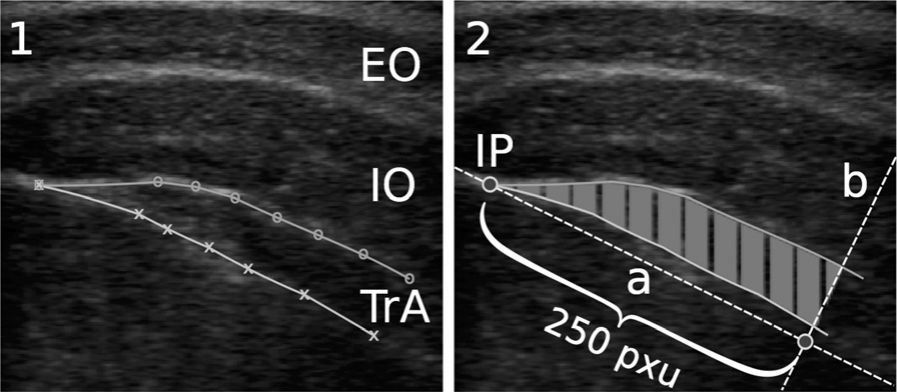
Measurement method. **1** Ultrasound image of the abdominal area on the APC, edited by manual set points. Indicates are the external oblique (EO), the internal oblique (IO) and the transverse abdominis (TrA). **2** Sectional area (grey band area) processed in MatLab® to determine the area between the superficial fascia (upper line with dotts) and the deep fascia (lower line with crosses) of the TrA (see data analysis)

### Statistics

SPSS v21 for Mac (IBM, New York) was used to calculate a two-way repeated-measurements ANOVA on the TrA thickness values with the factors “condition” (RC, SC, APC) and “time” (first, second and third measurement day). The results were corrected for violation of sphericity using the Greenhouse-Geisser method, and also for statistical bias using the Bonferroni method. Concerning the primary aim to compare the activation of the TrA between the conditions (RC, SC, APC) effect sizes (Cohen’s d, partial η^2^) were calculated and post-hoc tests were performed to test for significant differences on all three pairs of combinations.

As for the test-retest reliability, the intra-class correlation coefficient (ICC) and Cronbach’s α were determined with respect to the first and third measurement day (five days apart). The measurement days with the largest temporal distance were chosen to account for potential fluctuations in time.

## Results

### Quantification of TrA thickness

**Fig. 4:**
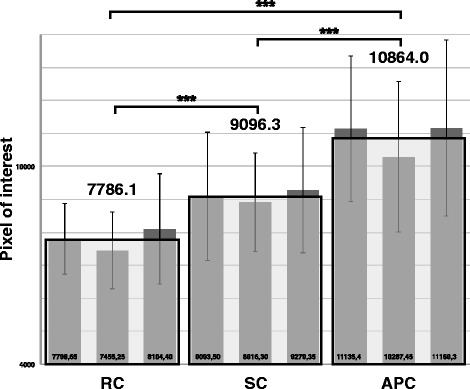
TrA thickness. TrA thickness (in pixel units pxu, see data analysis) on the three different conditions (resting condition = RC, sling condition = SC, abdominal press condition = APC). The bars represent the mean pxu for each measurement day on the different conditions (numerical values at the bottom of the bars). The gray boxes with the black frame represent the mean values over the three measurement days (numerical values over the gray boxes). The vertical lines in the bars indicate the standard deviation. ***: α < 0.001 indicates a (highly) significant difference between the mean values over the three measurement days (gray boxes with black frames)

The ANOVA revealed a significant main effect of the factor condition [F(2,38) = 47.82, *p* < 0.0001, η^2^ = 0.72], no significant main effect of the factor time [F(2.38) = 2.45, *p* = 0.1, η^2^ = 0.11), and no significant interaction effect [F(4,76) = 0.315, *p* = 0.867, η^2^ = 0.02]. The three post-hoc t-tests revealed significant differences showing that RC < SC (*p* < 0.001; η^2^ = 0.19; d = 0.96), SC < APC (*p* < 0.0001; η^2^ = 0.23; d = 1.10), and RC < APC (*p* < 0.0001; η^2^ = 0.53; d = 2.11) (Fig. 4).

### Test-retest reliability

The ICC and Cronbach’s α obtained the following values on the three different conditions: ICC = 0.71 (CI = [0.41,0.87]) and α = 0.83 on RC; ICC = 0.54 (CI = [0.12,0.79]) and α = 0.69 on SC; ICC = 0.29 (CI = [-0.19,0.65]) and α = 0.44 on APC, where “CI” indicates the 95 % confidence interval of the ICC.

## Discussion

The present study aimed to investigate the thickness of the *M. transversus abdominis* (TrA) as a measure for the muscle activation on three measurement conditions: resting condition (RC), sling condition (SC), and abdominal press condition (APC), using an ultrasound-based measurement method (USM). The results demonstrate that the sling exercise is associated with a significant increase of TrA thickness relative to the resting condition, and a significant decrease relative to the abdominal press condition. In line with other studies [4–7], we derive the activity of the TrA from its thickness. Theoretically, muscle thickness can change independently of muscle activation, *e.g.* during passive stretching or compression. However, as the subjects remained in a constant position throughout the exercise on all conditions, the TrA can only minimally be stretched or compressed by other abdominal muscles. On the SC, the TrA could additionally have been compressed by the viscera under the influence of gravity, which would have resulted in either a decrease or a reduced increase of TrA thickness relative to the SC. We measured a significant increase of TrA thickness, so that the latter effect, if present, did at least not conceal the increase of thickness due to muscle activation.

Until now there is no standardized measurement method to compare different muscle layers in the abdominal wall, mainly because there are no clearly defined biomarkers for the location and orientation of the transducer. For this study, we used a simple ultrasound-based quantification of muscle thickness for the sectional area enclosed by the superficial and the deep fascia of the TrA. A test-retest reliability analysis based on the first and third measurement day revealed an intra-class correlation coefficient (ICC) of 0.71 and a Cronbach’s α of 0.83 on the RC, which are sufficiently high values to accept the method as reliable on this measurement condition. However, on the other two conditions, the ICC and Cronbach’s α turned out to be relatively low (ICC = 0.54 and α = 0.69 on SC, ICC = 0.29 and α = 0.44 on APC), which indicates rather poor test-retest reliability and might be explained as follows:

First, the poor reliability on the SC and APC may be due to complications during the measurement, afflicting the reproducibility of the procedure. While it is relatively easy to place the transducer at the right spot on the abdomen when the subject is at rest (as on the RC), it is more difficult to place the transducer when the subject is suspended in the sling system (as on the SC), and also when the subject contracts his abdominal muscles with maximum voluntary contraction (as on the APC), so that the abdomen is deformed. Using a supportive device, *e.g.* a guiding belt, to fixate the transducer on the abdomen, might reduce these complications.

Second, if subjects contract their muscles in a reactive manner (as on the SC) or in a voluntary manner (as on the APC), it is difficult for them to reproduce the same level of contraction across several days [15]. It is especially difficult to voluntarily reproduce the same level of contraction, so the variability of measured muscle thickness is expected to be largest on the APC, which is what we have found.

Third, the ICC may additionally be impaired by potential neuromuscular adaptations induced by the muscle activation during the measurement sessions [16].

Despite these impairments on the reproducibility on the SC and APC resulting in low ICC values, we still found a statistically significant difference between TrA thickness values on the different conditions. We conclude that although the absolute value of TrA thickness assessed by the ultrasound-based measurement method used in this study may reliably be used only when the subject is at rest, the method is still reliable enough to differentiate between muscle thickness values on the individual conditions.

The test-retest reliability analysis was based on the first and third measurement day, thus on the largest available time interval (five days), to account for potential fluctuations of the thickness of the TrA over time.

We did not compare thickness values of abdominal muscles other than those of the TrA, because on the APC the intersection points of the OI and of the TrA could not be measured simultaneously using the USM method. It would also in this context be desirable to have a supportive device, *e.g.* a guiding belt, to take ultrasound images in a precisely defined transversal plane. We have conducted further studies (manuscripts in preparation) with an ultrasound guiding belt to improve the measurement procedure.

We think that the comparison of TrA activity on different exercise conditions may help to develop specific treatment strategies particularly for the management of low back pain. In this context, the effectiveness of sling exercises, especially the exercise mentioned above, should be clinically tested.

## Conclusions

We showed that during a specific exercise in a sling system, the thickness of the TrA was significantly increased as compared to the resting condition, but not as much as during abdominal press condition. We quantified the thickness of the TrA using an ultrasound-based measurement method. While the method may be used to detect differences in muscle thickness on the exercises applied in this study, it may be used to reliably detect absolute thickness values only when the subject is at rest.

## References

[1] Hodges PW (1999). Is there a role for transversus abdominis in lumbo-pelvic stability?. Man Ther.

[2] Himes ME, Selkow NM, Gore MA, Hart JM, Saliba SA (2012). Transversus abdominis activation during a side-bridge exercise progression is similar in people with recurrent low back pain and healthy controls. J Strength Cond Res.

[3] Reeve A, Dilley A (2009). Effects of posture on the thickness of transversus abdominis in pain-free subjects. Man Ther.

[4] Hodges PW, Pengel LHM, Herbert RD, Gandevia SC (2003). Measurement of muscle contraction with ultrasound imaging. Muscle Nerve.

[5] McMeeken JM, Beith ID, Newham DJ, Milligan P, Critchley DJ (2004). The relationship between EMG and change in thickness of transversus abdominis. Clin Biomechanics.

[6] Whittaker JL, Teyhen DS, Elliott JM, Cook K, Langevin HM, Dahl HH, Stokes M (2007). Rehabilitative ultrasound imaging: understanding the technology and its applications. J Orthop Sports Phys Ther.

[7] Teyhen DS (2006). Rehabilitative ultrasound imaging symposium: overview. J Orthop Sports Phys Ther.

[8] Ljunggren AE, Weber H, Kogstad O, Thom E, Kirkesola G (1997). Effect of Exercise on Sick Leave Due to Low Back Pain. Spine.

[9] Stuge B, Veierød MB, Lærum E, Vøllestad N (2004). The Efficacy of a Treatment Program Focusing on Specific Stabilizing Exercises for Pelvic Girdle Pain After Pregnancy. Spine.

[10] Vasseljen O, Fladmark AM (2010). Abdominal muscle contraction thickness and function after specific and general exercises: a randomized controlled trial in chronic low back pain patients. Man Ther.

[11] Lee J-S, Yang S-H, Koog Y-H, Jun H-J, Kim S-H, Kim K-J (2014). Effectiveness of sling exercise for chronic low back pain: a systematic review. J Phys Ther Sci.

[12] Misuri G, Colagrande S, Gorini M, Iandelli I, Mancini M, Duranti R, Scano G (1997). *In vivo* ultrasound assessment of respiratory function of abdominal muscles in normal subjects. Eur Respir J.

[13] Pinto RZ, Ferreira PH, Franco MR, Ferreira MC, Ferreira ML, Teixeira-Salmela LF, Oliviera VC, Maher C (2011). The effect of lumbar posture on abdominal muscle thickness during an isometric leg task in people with and without non-specific low back pain. Man Ther.

[14] Koppenhaver SL, Parent EC, Teyhen DS, Herbert JJ, Fritz JM (2009). The effect of averaging multiple trials on measurement error during ultrasound imaging of transversus abdominis and lumbar multifidus muscles in individuals with low back pain. J Orthop Sports Phys Ther.

[15] Dankaerts W, O’Sullivan PB, Burnett AF, Straker LM, Danneels LA (2004). Reliability of EMG measurements for trunk muscles during maximal and sub-maximal voluntary isometric contractions in healthy controls and CLBP patients. J Electromyographie Kinesiology.

[16] Tsao H, Hodges PW (2007). Immediate changes in feedforward postural adjustments following voluntary motor training. Exp Brain Res.

